# Impact of bariatric surgery in patients with stress urinary incontinence

**DOI:** 10.31744/einstein_journal/2021AO5701

**Published:** 2021-03-09

**Authors:** Antônio Flávio Silva Rodrigues, Fernando Korkes, Danielle de Sá Dantas Bezerra, Wilson Rodrigues de Freitas, Luís Gustavo Morato de Toledo

**Affiliations:** 1 Faculdade de Medicina do ABC Santo AndréSP Brazil Faculdade de Medicina do ABC, Santo André, SP, Brazil.; 2 Irmandade da Santa Casa de Misericórdia de São Paulo São PauloSP Brazil Irmandade da Santa Casa de Misericórdia de São Paulo, São Paulo, SP, Brazil.

**Keywords:** Urinary incontinence, Bariatric surgery, Menopause

## Abstract

**Objective:**

To examine epidemiologic, anthropometric and clinical variables associated with stress urinary incontinence in obese women, before and after bariatric surgery, and to identify predictive factors of stress urinary incontinence resolution.

**Methods:**

Prospective observational study with women enrolled in a bariatric surgery program between 2015 and 2016. Patients were assessed prior to and 6 months after bariatric surgery using the International Consultation on Incontinence Questionnaire-Urinary Incontinence Short Form, the Patient Global Impression of Improvement and the Visual Analogue Scale. Patient assessment also included physical examination and bladder stress tests.

**Results:**

A total of 43 women completed the study. There was a 72.7% reduction in stress urinary incontinence (p=0.021). Predictive factors for preoperative diagnosis of stress urinary incontinence included age (p=0.024) and abdominal waist circumference (p=0.048). Urinary symptoms improved after weight loss, especially nocturia (p=0.001) and stress urinary incontinence (p=0.026). Menopause was the most significant predictive factor for persistence of stress urinary incontinence within six months of bariatric surgery (p=0.046). Self-reported outcomes and scores obtained in the International Consultation on Incontinence Questionnaire-Urinary Incontinence Short Form, the Patient Global Impression of Improvement and the Visual Analogue Scale were associated with significant improvement (p=0.012, p=0.025, and p=0.002 respectively).

**Conclusion:**

Older women with larger waist circumference have a higher risk of developing stress urinary incontinence prior to bariatric surgery. Menopausal women are highly prone to persistent stress urinary incontinence, even after weight loss. Weight loss achieved through bariatric surgery improved stress urinary incontinence symptoms and mitigated related impacts on quality of life in the vast majority of women.

## INTRODUCTION

Stress urinary incontinence (SUI) is a prevalent disease. In a national survey, SUI was estimated to affect 30 million adults in the United States, with a prevalence of 54% in women and 15% in men.^(^^1^^)^ Stress urinary incontinence is a global health problem with considerable social and economic impacts, which affects patient quality of life and reduces productivity, leading to social isolation and depression.^(^^2^^)^

Obesity is a known risk factor for SUI. In epidemiologic studies, five-unit increments of body mass index (BMI) were associated with a 30% increase in severe urinary incontinence,^(^^3^^–^^5^^)^ especially SUI.^(^^6^^–^^8^^)^

Given obesity is a modifiable risk factor, weight loss within healthy limits may reduce SUI. Weight loss is a therapeutic intervention for patients with SUI.^(^^3^^)^ Low-calorie diets may result in weight loss (often around 7% to 10%) and improve SUI symptoms in obese women.^(^^9^^)^ Likewise, SUI improves in response to weight loss following bariatric surgery.^(^^4^^,^^10^^,^^11^^)^ Obese patients tend to experience a 53 to 56% reduction in BMI within six months of bariatric surgery and may lose up to 74% of their initial body weight within two years.^(^^12^^)^

## OBJECTIVE

To examine epidemiologic, anthropometric and clinical variables associated with stress urinary incontinence in obese women, before and after bariatric surgery, and to identify predictive factors of stress urinary incontinence resolution.

## METHODS

This prospective observational study was conducted at *Irmandade da Santa Casa de Misericórdia de São Paulo* São Paulo (SP), Brazil, in collaboration with *Faculdade de Medicina do ABC*, Santo André (SP), Brazil, undergraduate/graduate programs. The sample comprised 46 women enrolled in the Bariatric Surgery Program offered by a philanthropic institution located in São Paulo, between 2015 and 2016.

This study was approved by the Institutional Review Board and Ethics Committee (protocol 2.076.379; approval CAAE: 56382216.5.0000.5479). Patients who did not accept to participate or failed to complete the protocol (n=2) were excluded, as well as one patient who died after surgery.

Remaining patients met the following institutional eligibility criteria for bariatric surgery: BMI higher than 40kg/m^2^ or 35 to 40kg/m^2^ and obesity-related comorbidities, age between 17 to 65 years, motivation to undergo surgery, possibility of life-long follow-up, sufficient cognitive ability to understand the procedure, lack of drug or alcohol addictions and multidisciplinary preoperative evaluation – psychology, psychiatry, nutrition, physical therapy and endocrinology.

Patients were enrolled in the study prior to hospital admission and were allowed to decide whether to participate on their own free will. Patients who consented to participate after being duly informed were asked to answer questionnaires and examined. Three women failed to properly fill out the questionnaires and were excluded from the study.

The sample included women scheduled for bariatric surgery who agreed to participate. Patients were examined in the preoperative unit the day before bariatric surgery, then within six months of surgery. All women were submitted to the Y-en-Roux gastric bypass bariatric procedure.

Examinations comprised history taking, physical examination (including bladder stress test) and the administration of the International Consultation on Incontinence Questionnaire-Urinary Incontinence Short Form (ICIQ-UI SF),^(^^13^^)^ Patient Global Impression of Improvement (PGI-I)^(^^14^^)^ and Visual Analogue Scale (VAS) for satisfaction.^(^^15^^)^ All patients were evaluated by the same urologist. Selected variables included age, ethnicity, weight, height, BMI, waist circumference (cm) and gynecologic and obstetric history (including menopause, parity, miscarriages and newborn weight). Comorbidities and some habits, such as asthma, diabetes, hypertension and smoking were also investigated.

Urinary symptoms were actively investigated. These included storage symptoms such as SUI, urge, urge incontinence, mixed incontinence and nocturia, and voiding symptoms such as intermittent voiding, weak stream, maneuvers to reduce genital prolapse during micturition, and dyspareunia.

Stress urinary incontinence symptoms and their impact on patient´s quality of life were assessed using the ICIQ-UI SF validated for the Portuguese language. After this initial evaluation, urine leakage during the physical examination was interpreted as positive stress test. All women were examined in the supine and the orthostatic position. Valsalva maneuvers and the cough stress test were performed. Absence of urine leakage and a minimum of 200mL of voided urine after the examination were interpreted as negative test.

Only patients with incontinence complaints and leakage during physical examination received a diagnosis of SUI.^(^^16^^)^ Pelvic prolapses were classified using to the Pelvic Organ Prolapse Quantification (POP-Q) system.^(^^17^^)^

The second evaluation, conducted within 6 months of surgery, followed the same sequence. Resolution of symptoms was assessed using the PGI-I and the VAS for satisfaction. Patient satisfaction regarding improvement of urinary symptoms was rated zero to ten. Subjective success was defined as patient perception of improvement (very much better or much better, PGI-I scores one and two, respectively) and VAS scores of eight or higher. Objective success was defined as absence of leakage during stress tests conducted with a minimum of 200mL of urine in the bladder.

After preliminary analysis of results, three groups were created according to SUI: Group A, with women without preoperative SUI; Group B, with women with preoperative diagnosis of SUI, who achieved postoperative resolution and Group C, women with preoperative SUI who did not achieve resolution.

Statistical analysis was conducted using SPSS, version 13.0 (SPSS for Mac OS X, SPSS, Inc., Chicago, Illinois). Intergroup comparisons were performed using the Pearson's χ^2^ test and Analysis of Variance (Anova). Statistical significance was set at p<0.05. Following adjustment for variables, selection was performed according to the Akaike information criterion (AIC).

## RESULTS

**Figure 1:**
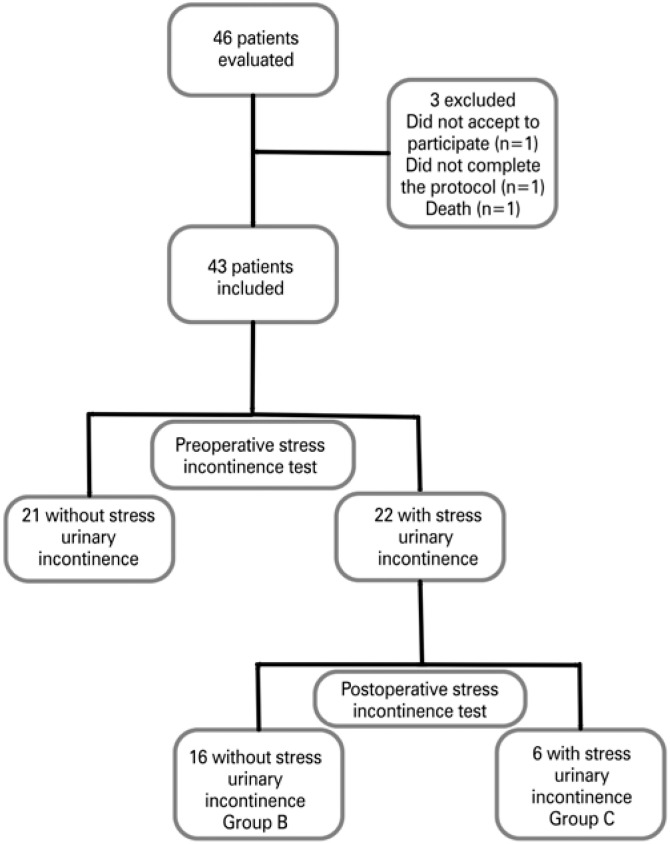
Consolidated Standards of Reporting Trials flowchart of patients over the course of the experimental period, from 2015 to 2016

The initial sample comprised 46 patients. Of these, three were excluded. The final sample comprised 43 women with a mean age of 41.4±9.8 years. Mean BMI decreased 25.1%, from 45.71±5.80kg/m^2^ preoperatively to 34.32±3.48kg/m^2^, postoperatively. Overall, 22 (51.2%) patients had a preoperative diagnosis of SUI. There was a 72.7% reduction in SUI within 6 months (p=0.021). However, six women did not achieve SUI resolution. The Consolidated Standards of Reporting Trials (CONSORT) flowchart of patients is shown in figure 1.

Groups A comprised 21 women (48%). Groups B and C comprised 16 and six women (72.7% and 27.3% of women with preoperative incontinence, respectively). Demographics of the sample according to preoperative SUI status are shown in table 1.

**Table 1:** Associations between demographics and stress urinary incontinence before bariatric surgery

Variable		No SUI (n=21)	SUI (n=22)	p value*
Initial weight, kg		116.1±14.6	123.1±21.9	0.488
Ethnicity				0.242
	White	62 (13)	59 (13)	
	Black	19 (4)	4.5 (1)	
	Non-white	19 (4)	36.5 (8)	
Age, years		36.9±8.23	45.7±9.28	0.002
Height, cm		161.0±6.66	161.4±7.08	0.884
BMI, kg/m^2^		44.4±4.16	47.0±6.90	0.189
Waist circumference, cm		124.0±10.7	135.0±19.6	0.041
Heaviest newborn weight, kg		3.43±0.73	4.07±0.85	0.056
Parity		1.23±1.01	2.09±2.18	0.397
Vaginal deliveries		0.52±0.83	1.181±1.33	0.155
C-section deliveries		0.61±0.72	1.27±1.21	0.293
Miscarriages		0.09±0.29	0.50±0.71	0.051
Menopause		14.3 (3)	40.9 (9)	0.061
Hypertension		47.6 (10)	59.1 (13)	0.452
Diabetes		23.8 (5)	31.8 (7)	0.559
Smoking		14.3 (3)	36.6 (8)	0.107
Asthma		0	18.1 (4)	0.993
Genital prolapse		0	18.1 (4)	0.993

Results expressed as mean±standard deviation or n (%).

* Pearson's χ^2^ test and Mann-Whitney test.

SUI: stress urinary incontinence; BMI: body mass index.

Initial weight, ethnicity, height, BMI, larger newborns, parity, number of vaginal or C-section deliveries and miscarriages, menopause, hypertension, diabetes, smoking, asthma and genital prolapse did not differ among groups according to SUI status. Participants in the SUI group were older (p=0.024) and had larger waist circumference (p=0.048).

Prior to surgery, each additional year of age increased the risk of SUI by 11.8% (1.11; 95% of confidence interval – 95%CI: 1.04-1.20; p=0.009), whereas each one centimeter of gain in waist circumference increased the risk of SUI by 5.7% (1.05; 95%CI: 1.01-1.11; p=0.05).

Table 2 provides a comparison of symptoms prior to and after bariatric surgery.

**Table 2:** Symptoms before to and after bariatric surgery

Symptoms	Preoperative (n=43)	Postoperative (n=43)	p value*
Stress UI	22 (51)	11 (25)	0.026
Urge UI	20 (46)	12 (27)	0.118
Mixed UI	20 (46)	11 (25)	0.072
Nocturia	15 (34)	2 (4)	0.001
Voiding symptoms	14 (32)	6 (14)	0.074
Dyspareunia	4 (9)	0 (0)	0.124
ICIQ-UI SF	9.33 (21 points)	3.75 (21 points)	0.012

Results expressed as n (%).

* Pearson's χ^2^ test.

UI: urinary incontinence; ICIQ-UI SF: International Consultation on Incontinence Questionnaire-Urinary Incontinence Short Form.

Urinary symptoms improved after weight loss, particularly nocturia (p=0.001) and SUI (p=0.026).

Table 3 shows results of univariate and multivariate regressions employed to investigate factors associated with SUI persistence after surgery. Multivariate modeling was performed according to the AIC criteria for variable selection.

**Table 3:** Univariate and multivariate models for stress urinary incontinence persistence after surgery

Variable	Univariate regression analysis		Multivariate regression analysis	
	OR (95%CI)	p value	OR (95%CI)	p value
Difference in weight	1.04 (0.95-1.14)	0.339		
Age	1.27 (1.00-1.61)	0.041		
Height	0.96 (0.83-1.10)	0.595		
Difference in BMI	1.15 (0.90-1.46)	0.249		
Difference in waist circumference	0.97 (0.87-1.08)	0.587		
Heaviest newborn weight	1.68 (0.70-4.01)	0.242		
Parity	1.46 (0.87-2.44)	0.147		
Vaginal deliveries	2.51 (1.08-5.84)	0.031	6.68 (0.87-91.74)	0.157
C-section deliveries	0.63 (0.25-1.53)	0.310		
Miscarriages	1.51 (0.43-5.25)	0.512		
Menopause	15.0 (1.32-169.86)	0.029	12.71 (1.81-167.3)	0.046
Hypertension	16.46 (0.79-341.3)	0.070		
Diabetes	3.00 (0.42-21.29)	0.272		
Smoking	2.20 (0.32-14.97)	0.420		
Asthma	3.50 (0.36-33.30)	0.276		

OR: odds ratio; 95%CI: 95% confidence interval; BMI: body mass index.

Stress urinary incontinence persistence was significantly associated with age, vaginal deliveries and menopause (p=0.041, p=0.031 and p=0.029, respectively; univariate analysis). Multivariate analysis including vaginal deliveries and menopause in the model revealed significant associations between menopause and SUI persistence. SUI persistence was 12.7 times more likely in menopausal relative to non-menopausal women (OR: 12.72; 95%CI: 1.81-167.32, p=0.046).

Table 4 provides a comparative analysis of postoperative VAS, PGI-I and ICIQ-UI SF scores obtained by participants with a preoperative diagnosis of SUI, who achieved postoperative resolution (Group B) and those who did not achieve resolution (Group C).

**Table 4:** Comparisons between Group B (preoperative diagnosis of stress urinary incontinence with postoperative resolution) and C (preoperative diagnosis of stress urinary incontinence without postoperative resolution)

Variable	Group B	Group C	p value
VAS (8-10)	100 (16)	33.3 (2)	0.002
PGI-I (1-2)	87.5 (14)	33.3 (2)	0.025
ICIQ-UI SF	1.65±2.5	9.33±2.87	0.012

Results expressed as n (%) or mean±standard deviation.

* Pearson's χ^2^ test.

VAS: Visual Analogue Scale; PGI-I: Patient Global Impression of Improvement; ICIQ-UI SF: International Consultation on Incontinence Questionnaire-Urinary Incontinence Short Form.

The analysis of self-reported outcomes revealed better VAS (p=0.002), PGI-I (p=0.025) and ICIQ-UI SF (p=0.012) scores in Group B than in Group C. The frequency of subjective success according to VAS (scores eight to ten) was higher in Group B (100.0%) than in Group C (33.3%). Likewise, PGI-I scores one and two (very much better or much better) were more common in Group B (87.5%) than in Group C (33.3%). Finally, mean postoperative ICIQ-UI SF scores were significantly lower in Group B than in Group C (1.65 and 9.33 respectively).

## DISCUSSION

The association between obesity and urinary incontinence is well known.^(^^1^^,^^4^^,^^10^^,^^11^^,^^18^^)^ As expected, 22 out of 43 women (51.1%) in this study were diagnosed with SUI. Weight loss is a well-established therapeutic approach for SUI and is recommended as a feasible and interesting strategy for these women.^(^^3^^)^ Previous studies have shown that modest (5% to 10%) weight loss translates into significant benefits for obese women with urinary incontinence.^(^^9^^)^ Women in this cohort experienced a weight loss of 25.1% in the first 6 months after bariatric surgery. Weight loss tends to be more dramatic during this period. However, operated patients can lose up to 74% of their initial body weight over the course of two years.^(^^12^^)^ This study contributed important findings. First, participants in the SUI group were older (p=0.024) and had larger waist circumference (p=0.048). Each additional year of age increased the risk of SUI by 11.8% (p=0.009). For every one centimeter of gain in waist circumference, the risk of developing SUI prior to surgery increased by 5.7% (p=0.05). The prevalence and severity of urinary incontinence increase with age. Population studies reveal a 30% to 40% prevalence of urinary incontinence among middle-aged women and a higher prevalence (up to 57%) in older women.^(^^2^^,^^4^^)^However, studies controlling for other comorbidities suggested age is not an independent risk factor for incontinence. Higher levels of central adiposity and resultant increased abdominal pressure may be significant risk factors for SUI. For example, combined analysis of BMI and waist circumference in 2,702 women aged 42 to 52 years revealed an increased risk for prevalence of SUI with every one centimeter of gain in waist circumference (OR=1.04; 95%CI: 1.02-1.06), but not with unit increments of BMI (OR=0.99; 95%CI: 0.95-1.04).^(^^5^^)^

Secondly, SUI decreased significantly within 6 months of bariatric surgery. In this cohort, 51.1% of women had a preoperative diagnosis of SUI. However, only 14% failed to achieve incontinence resolution within 6 months of surgery (p=0.021), a resolution rate of 72.7%. Urinary symptoms also improved, particularly SUI (51% to 25%; p=0.026) and nocturia (34% to 4%; p=0.001). Findings of this study are congruent with those reported by other authors (SUI resolution rate ranging from 80 to 92% after significant weight loss).^(^^8^^,^^10^^,^^11^^)^

Thirdly, patient quality of life improved. International Consultation on Incontinence Questionnaire-Urinary Incontinence Short Form scores decreased by 85.3% in women achieving SUI resolution (7.68 points lower relative to women who did not achieve resolution on average). Weight loss has a significant impact on quality of life and correlations with urodynamic findings have been reported.^(^^10^^,^^19^^)^ Patient Global Impression of Improvement and VAS for satisfaction scores revealed improvement of reported symptoms (p=0.025 and p=0.002), suggesting improvements in overall well-being, not only due to weight loss, but also to indirect gains such as SUI resolution.^(^^14^^,^^16^^)^ The opposite is also true: quality of life/satisfaction scores were 2.5 points lower (p=0.004) among women who did not achieve SUI resolution.^(^^15^^)^ Therefore, occurrence of SUI after weight loss is expected to promote even greater improvement in quality of life.

Fourthly, important predictors of SUI outcomes after weight loss were identified in this study. Persistence of SUI was significantly associated with age, vaginal delivery and menopause (p=0.041, p=0.031 and p=0.029, respectively; univariate analysis), but menopause was the most significant predictor of SUI persistence within six months of bariatric surgery (multivariate analysis). Persistence of SUI after surgery was 2.7 times more likely in menopausal than in non-menopausal women (p=0.046). Menopause was associated with an increased risk of SUI. However, age and menopause are closely related, and age is a well-known factor for SUI: the risk of SUI is 1.8-fold higher in women aged 50 years relative to women aged 40 years.^(^^4^^)^Age- and menopause-related persistence of SUI after weight loss results from low serum testosterone levels and sarcopenia. Fast and excessive weight loss may lead to sarcopenia. Low serum testosterone levels are associated with a higher risk of incontinence in women, given the contribution of pelvic muscles to urethral support and the anabolic effects of androgens on skeletal muscle.^(^^20^^,^^21^^)^ Testosterone replacement may improve the prognosis of SUI patients. However, associations between SUI and low serum testosterone levels have not been well-established to date. Age may also affect pelvic floor integrity, leading to urethral hypermobility and intrinsic sphincter deficiency in response to loss of muscle tone and thinning of the urethral mucosa.

This may impair urethral closure, leading to urine leakage during episodes of increased abdominal pressure (sneezing, coughing etc.).^(^^16^^,^^17^^,^^21^^)^

Finally, this study failed to reveal correlations between the magnitude of weight loss and postoperative SUI (p=0.33). Stress urinary incontinence appears to be multifactorial in obese women. Similar findings have been reported in previous studies with longer follow-up.^(^^10^^,^^11^^)^

This study has some limitations. Sample size was relatively small and follow-up was short. However, findings of this study provide insights into how to gauge expectations prior to bariatric surgery and may assist in the identification of candidates who are more likely to require additional procedures to control SUI.

## CONCLUSION

In conclusion, older women with larger waist circumference have a higher risk of developing stress urinary incontinence prior to bariatric surgery. This type of urinary incontinence tends to persist in menopausal women in spite of weight loss. Weight loss achieved through bariatric surgery improves stress urinary incontinence symptoms, and reduces the impacts on quality of life in the vast majority of women.
