# Supplementary material for: Impact of bariatric surgery in patients with stress urinary incontinence
Source: Einstein (Sao Paulo). 2021 Mar 9;19:eAO5701. doi: 10.31744/einstein_journal/2021AO5701 (PMC7942840; doi:10.31744/einstein_journal/2021AO5701)
Supplement: Translation

# Translation: Impacto da cirurgia bariátrica em pacientes com incontinência urinária de esforço

**Authors:** Antônio Flávio Silva Rodrigues, Fernando Korkes, Danielle de Sá Dantas Bezerra, Wilson Rodrigues de Freitas, Luís Gustavo Morato de Toledo

**Affiliations:** 1 Santo AndréSP Brasil Faculdade de Medicina do ABC, Santo André, SP, Brasil.; 2 São PauloSP Brasil Irmandade da Santa Casa de Misericórdia de São Paulo, São Paulo, SP, Brasil.

**Keywords:** Incontinência urinária, Cirurgia bariátrica, Menopausa

## Abstract

**Objetivo:**

Examinar as variáveis epidemiológicas, antropométricas e clínicas associadas à incontinência urinária de esforço em mulheres obesas antes e após a cirurgia bariátrica e identificar fatores preditivos da resolução desse tipo de incontinência.

**Métodos:**

Estudo observacional prospectivo com mulheres de um programa de cirurgia bariátrica, realizado entre 2015 e 2016. As pacientes responderam ao *International Consultation on Incontinence Questionnaire-Urinary Incontinence Short Form*, à *Clinical Global Impression-Improvement* e à Escala Visual Analógica, sendo submetidas ao exame físico e ao teste de incontinência antes e 6 meses após cirurgia bariátrica.

**Resultados:**

Completaram o estudo 43 mulheres. Houve redução de 72,7% na incontinência urinária de esforço (p=0,021). Fatores preditivos para o diagnóstico pré-operatório da incontinência urinária incluíram idade (p=0,024) e circunferência abdominal (p=0,048). Todos os sintomas urinários demonstraram melhora após perda de peso, notadamente noctúria (p=0,001) e incontinência urinária de esforço (p=0,026). A menopausa foi o fator mais crítico para predizer a persistência da incontinência urinária de esforço 6 meses após a cirurgia bariátrica (p=0,046). Os resultados relatados do *International Consultation on Incontinence Questionnaire-Urinary Incontinence Short Form*, da *Patient Global Impression of Improvement* e da Escala Visual Analógica tiveram melhora significativa (p=0,012, p=0,025, p=0,002, respectivamente).

**Conclusão:**

Mulheres idosas e com maior circunferência abdominal têm maior risco de desenvolver incontinência urinária de esforço antes da cirurgia. Mulheres na menopausa são fortemente propensas a persistir com a incontinência urinária de esforço, mesmo após a perda de peso. A perda de peso após a cirurgia bariátrica melhora os sintomas de incontinência urinária de esforço e seus impactos na qualidade de vida na maioria das mulheres.

## INTRODUÇÃO

A incontinência urinária de esforço (IUE) é uma doença prevalente. Em uma enquete nacional, foi estimado que ela afeta 30 milhões de adultos nos Estados Unidos, com prevalência de 54% em mulheres e de 15% em homens.^(^^1^^)^ Trata-se de um problema global de saúde, com impactos sociais e econômicos consideráveis, que afeta a qualidade de vida e reduz a produtividade, levando ao isolamento e à depressão.^(^^2^^)^

A obesidade é um fator de risco conhecido para IUE. Em estudos epidemiológicos, incrementos de cinco unidades no índice de massa corporal (IMC) foram associados a aumento de 30% da incontinência urinária grave,^(^^3^^–^^5^^)^ principalmente IUE.^(^^6^^–^^8^^)^

Como a obesidade é um fator de risco modificável, a perda de peso dentro de limites saudáveis pode reduzir a IUE. A perda de peso é uma intervenção terapêutica para pacientes com IUE.^(^^3^^)^ Dietas de baixo teor calóricopodem resultar em perda de peso (geralmente em torno de 7% a 10%) e na melhora dos sintomas de IUE em mulheres obesas.^(^^9^^)^ Analogamente, a IUE melhora em reposta à perda de peso após a cirurgia bariátrica.^(^^4^^,^^10^^,^^11^^)^ Pacientes obesos tendem a apresentar redução de 53% a 56% do IMC dentro de 6 meses após a cirurgia bariátrica e podem perder até 74% de seu peso inicial dentro de 2 anos.^(^^12^^)^

## OBJETIVO

Examinar as variáveis epidemiológicas, antropométricas e clínicas associadas à incontinência urinária de esforço em mulheres obesas antes e após a cirurgia bariátrica e identificar fatores preditivos da resolução desse tipo de incontinência.

## MÉTODOS

Este estudo observacional prospectivo foi realizado na Irmandade da Santa Casa de Misericórdia de São Paulo, em São Paulo (SP), em colaboração com a Faculdade de Medicina do ABC, Santo André (SP), pelos programas de graduação e pós-graduação. A amostra incluiu 46 mulheres inscritas no Programa de Cirurgia Bariátrica oferecido por uma instituição filantrópica localizada em São Paulo, entre 2015 e 2016.

O estudo foi aprovado pelo Comitê de Ética da instituição, com parecer 2.076.379; aprovação CAAE: 56382216.5.0000.5479. As pacientes que não concordaram em participar ou não completaram o protocolo (n=2) foram excluídas, assim como uma que foi a óbito após a cirurgia.

As pacientes restantes atenderam aos seguintes critérios institucionais de elegibilidade: IMC acima de 40kg/m^2^ ou de 35 a 40kg/m^2^ e comorbidades relacionadas à obesidade, idade entre 17 e 65 anos, motivação para passar pela cirurgia, possibilidade de seguimento vitalício, capacidade cognitiva suficiente para entender o procedimento, ausência de vício em drogas ou álcool e avaliação pré-operatória multidisciplinar (psicologia, psiquiatria, nutrição, fisioterapia e endocrinologia).

As pacientes foram incluídas no estudo antes da internação hospitalar e optaram voluntariamente por participar ou não. As que concordam em participar após o fornecimento das devidas informações foram convidadas a responder a questionários, tendo sido examinadas. Três mulheres que não preencheram corretamente os questionários foram excluídas do estudo.

A amostra incluiu mulheres aguardando cirurgia bariátrica que concordaram em participar. As pacientes foram examinadas na unidade pré-operatória no dia anterior à cirurgia bariátrica e 6 meses após a cirurgia. Todas elas foram submetidas ao procedimento bariátrico de *bypass* gástrico em Y de Roux.

Os exames incluíram anamnese, exame físico com teste de estresse da bexiga e administração dos questionários *International Consultation on Incontinence Questionnaire-Urinary Incontinence Short Form* (ICIQ-UI SF),^(^^13^^)^ *Patient Global Impression of Improvement* (PGI-I)^(^^14^^)^ e Escala Visual Analógica (EVA) para satisfação.^(^^15^^)^ Todas as pacientes foram avaliadas pelo mesmo urologista. As variáveis selecionadas foram idade, etnia, peso, altura, IMC, circunferência abdominal (cm) e histórico ginecológico e obstétrico (incluindo menopausa, paridade, abortos e peso do recém-nascido). Comorbidades e hábitos como asma, diabetes, hipertensão e tabagismo também foram investigados.

Os sintomas urinários foram investigados ativamente e incluíram manifestações relacionadas ao armazenamento, como IUE, urgência, incontinência de urgência, incontinência mista e noctúria, além de sintomas urinários, como jato intermitente, fluxo fraco, manobras para diminuir o prolapso genital durante a micção e dispareunia.

O ICIQ-UI SF validado para o português foi utilizado para avaliar os sintomas de IUE e o respectivo impacto sobre a qualidade de vida das pacientes. Após a avaliação inicial, a perda de urina durante o exame físico caracterizou resultado positivo no teste de estresse. Todas as mulheres foram examinadas na posição supinada e ortostática. As manobras de Valsalva e de tosse foram realizadas. A ausência de perda de urina com pelo menos 200mL de jato após o exame foi interpretada como resultado negativo no teste.

O diagnóstico de IUE foi limitado a pacientes com queixas de incontinência e perda de urina durante o exame físico.^(^^16^^)^ Os prolapsos pélvicos foram classificados de acordo com o sistema *Pelvic Organ Prolapse Quantification* (POP-Q).^(^^17^^)^

A segunda avaliação, realizada 6 meses após a cirurgia, seguiu a mesma sequência. A resolução dos sintomas foi avaliada empregando-se o PGI-I e a EVA para satisfação. A satisfação das pacientes quanto à melhora dos sintomas urinários foi graduada de zero a dez. O sucesso subjetivo foi definido como percepção de melhora por parte das pacientes (extremamente melhor ou muito melhor, escores PGI-I um e dois, respectivamente) e escores de oito ou superiores na EVA. O sucesso objetivo foi definido como ausência de perda de urina durantes os testes de estresse conduzidos com um mínimo de 200mL de urina na bexiga.

Após a análise preliminar dos resultados, três grupos foram criados, de acordo com a IUE: Grupo A, mulheres sem IUE pré-operatória; Grupo B, de mulheres com IUE pré-operatória e com resolução pós-operatória e Grupo C, de mulheres com IUE pré-operatória e sem resolução.

A análise estatística foi realizada por meio de software SPSS, versão 13.0 (SPSS para Mac OS X, SPSS, Inc., Chicago, Illinois). Os grupos foram comparados empregando-se o teste do χ^2^ de Pearson e a Análise de Variância (Anova). O nível de significância estabelecido foi de p<0,05. Após o ajuste das variáveis, a seleção foi realizada com base no critério de informação de Akaike (AIC).

## RESULTADOS

A amostra inicial incluiu 46 pacientes. Destas, três foram excluídas, restando uma amostra de 43 mulheres com média de idade de 41,4±9,8 anos. O IMC médio diminuiu em 25,1%, passando de 45,71±5,80kg/m^2^, antes da cirurgia, para 34,32±3,48kg/m^2^, no pós-operatório. Ao todo, 22 (51,2%) pacientes foram diagnosticadas com IUE antes da cirurgia. A ocorrência de IUE caiu 72,7% dentro de 6 meses (p=0,021). Entretanto, a incontinência persistiu em seis mulheres. O fluxograma *Consolidated Standards of Reporting Trials* (CONSORT) das pacientes encontra-se ilustrado na figura 1.

**Figura 1:** Fluxograma *Consolidated Standards of Reporting Trials* das pacientes ao longo do período experimental, de 2015 a 2016

O Grupo A incluiu 21 mulheres (48%). Os Grupos B e C incluíram 16 e seis mulheres (72,7% e 27,3% das mulheres com incontinência pré-operatória, respectivamente). Os parâmetros demográficos da amostra, de acordo com as características de IUE pré-operatória, encontram-se descritos na tabela 1.

**Tabela 1:** Associações entre parâmetros demográficos e incontinência urinária de esforço antes da cirurgia bariátrica

Variável		Sem IUE (n=21)	IUE (n=22)	Valor de p*
Peso inicial, kg		116,1±14,6	123,1±21,9	0,488
Etnia				0,242
	Branca	62 (13)	59 (13)	
	Negra	19 (4)	4,5 (1)	
	Não branca	19 (4)	36,5 (8)	
Idade, anos		36,9±8,23	45,7±9,28	0,002
Altura		161,0±6,66	161,4±7,08	0,884
IMC, kg/m^2^		44,4±4,16	47,0±6,90	0,189
Circunferência abdominal, cm		124,0±10,7	135,0±19,6	0,041
Maior peso do recém- nascido, kg		3,43±0,73	4,07±0,85	0,056
Paridade		1,23±1,01	2,09±2,18	0,397
Partos vaginais		0,52±0,83	1,181±1,33	0,155
Partos por cesariana		0,61±0,72	1,27±1,21	0,293
Abortos		0,09±0,29	0,50±0,71	0,051
Menopausa		14,3 (3)	40,9 (9)	0,061
Hipertensão		47,6 (10)	59,1 (13)	0,452
Diabetes		23,8 (5)	31,8 (7)	0,559
Tabagismo		14,3 (3)	36,6 (8)	0,107
Asma		0	18,1 (4)	0,993
Prolapso genital		0	18,1 (4)	0,993

Resultados expressos por média±desvio-padrão ou n (%).

* Testes do χ^2^ de Pearson e teste de Mann-Whitney.

IUE: incontinência urinária de esforço; IMC: índice de massa corporal.

As variáveis peso inicial, etnia, altura, IMC, maior peso do recém-nascido, paridade, número de partos vaginais ou por cesariana e abortos, menopausa, hipertensão, diabetes, tabagismo, asma e prolapso genital não diferiram entre os grupos de acordo com as manifestações de IUE. A participantes do grupo IUE eram mais velhas (p=0,024) e tinham circunferência abdominal maior (p=0,048).

Antes da cirurgia, cada ano adicional de idade aumentou o risco de IUE em 11,8% (1,11); intervalo de confiança de 95% – IC95%: 1,04-1,20; p=0,009), enquanto cada centímetro adicional de circunferência abdominal aumentou o risco de IUE em 5,7% (1,05; IC95%: 1,01-1,11; p=0,05).

A tabela 2 apresenta uma comparação dos sintomas antes e depois da cirurgia bariátrica.

**Tabela 2:** Sintomas antes e depois da cirurgia bariátrica

Sintomas	Pré-operatório (n=43)	Pós-operatório (n=43)	Valor de p*
IU de esforço	22 (51)	11 (25)	0,026
IU de urgência	20 (46)	12 (27)	0,118
IU mista	20 (46)	11 (25)	0,072
Noctúria	15 (34)	2 (4)	0,001
Sintomas de micção	14 (32)	6 (14)	0,074
Dispareunia	4 (9)	0 (0)	0,124
ICIQ-UI SF	9,33 (21 pontos)	3,75 (21 pontos)	0,012

Resultados expressos como n (%).

* Teste do χ^2^ de Pearson.

IU: incontinência urinária; ICIQ-UI SF: *International Consultation on Incontinence Questionnaire-Urinary Incontinence Short Form.*

Os sintomas urinários melhoraram após a perda de peso, principalmente a noctúria (p=0,001) e a IUE (p=0,026).

A tabela 3 mostra os resultados das regressões univariada e multivariadas empregadas para investigar fatores associados à persistência da IUE após a cirurgia. A modelagem multivariada foi realizada de acordo com os critérios AIC para seleção de variáveis.

**Tabela 3:** Modelos de análise univariada e multivariada empregados para persistência da incontinência urinária de esforço após a cirurgia

Variável	Análise de regressão univariada		Análise de regressão multivariada	
	RC (IC95%)	Valor de p	RC (IC95%)	Valor de p
Diferença de peso	1,04 (0,95-1,14)	0,339		
Idade	1,27 (1,00-1,61)	0,041		
Altura	0,96 (0,83-1,10)	0,595		
Diferença de IMC	1,15 (0,90-1,46)	0,249		
Diferença de circunferência abdominal	0,97 (0,87-1,08)	0,587		
Maior peso de recém-nascido	1,68 (0,70-4,01)	0,242		
Paridade	1,46 (0,87-2,44)	0,147		
Partos vaginais	2,51 (1,08-5,84)	0,031	6,68 (0,87-91,74)	0,157
Partos por cesarianas	0,63 (0,25-1,53)	0,310		
Abortos	1,51 (0,43-5,25)	0,512		
Menopausa	15,0 (1,32-169,86)	0,029	12,71 (1,81-167,3)	0,046
Hipertensão	16,46 (0,79-341,3)	0,070		
Diabetes	3,00 (0,42-21,29)	0,272		
Tabagismo	2,20 (0,32-14,97)	0,420		
Asma	3,50 (0,36-33,30)	0,276		

RC: razão de chances; IC95%: intervalo de confiança de 95%; IMC: índice de massa corporal.

A persistência da IUE mostrou-se significativamente associada a idade, parto vaginal e menopausa (p=0,041, p=0,031 e p=0,029, respectivamente; análise univariada). A análise multivariada com a inclusão de partos vaginais e menopausa no modelo revelou associações significantes entre menopausa e persistência da IUE, sendo a persistência 12,7 vezes mais provável em mulheres que estavam na menopausa do que nas demais (RC: 12,72; IC95%: 1,81-167,32; p=0,046).

A tabela 4 mostra a análise comparativa dos escores obtidos na EVA, no PGI-I e no ICIQ-UI SF pelas participantes diagnosticadas com IUE pré-operatória, que atingiram a resolução (Grupo B) e as que não atingiram a resolução (Grupo C).

**Tabela 4:** Comparações entre os Grupos B (incontinência urinária de esforço pré-operatória com resolução pós-operatória) e C (incontinência urinária de esforço sem resolução pós-operatória)

Variável	Grupo B	Grupo C	Valor de p
EVA (8-10)	100 (16)	33,3 (2)	0,002
PGI-I (1-2)	87,5 (14)	33,3 (2)	0,025
ICIQ-UI SF	1,65±2,5	9,33±2,87	0,012

Resultados expressos por n (%) ou média±desvio-padrão.

* Teste do χ^2^ de Pearson.

EVA: Escala Visual Analógica; PGI-I: *Patient Global Impression of Improvement*; ICIQ-UI SF: *International Consultation on Incontinence Questionnaire-Urinary Incontinence Short Form*.

A análise dos desfechos autorrelatados revelou melhora nos escores obtidos na EVA (p=0,002), no PGI-I (p=0,025) e no ICIQ-UI SF (p=0,012) nos Grupos B e C. A frequência de sucesso subjetivo segundo a EVA (escores oito a dez) foi mais alta no Grupo B (100,0%) do que no Grupo C (33,3%). De modo semelhante, escores PGI-I um e dois (extremamente melhor e muito melhor) foram mais frequentes no Grupo B (87,5%) do que no C (33,3%). Finalmente, a média dos escores ICIQ-UI SF pós-operatórios foi significativamente mais baixa no Grupo B do que no C (1,65 e 9,33, respectivamente).

## DISCUSSÃO

A associação entre a obesidade e a incontinência urinária é bem conhecida.^(^^1^^,^^4^^,^^10^^,^^11^^,^^18^^)^ Conforme esperado, 22 de 43 mulheres (51,1%) neste estudo foram diagnosticadas com IUE. A perda de peso constitui abordagem terapêutica bem-estabelecida para a IUE e é recomendada como estratégia viável e interessante para essas mulheres.^(^^3^^)^ Estudos prévios mostraram que perdas modestas de peso (de 5% a 10%) se traduzem em benefícios importantes para mulheres obesas com incontinência urinária.^(^^9^^)^ Nesta coorte, as mulheres apresentaram perda de peso de 25,1% nos primeiros 6 meses após a cirurgia bariátrica. A perda de peso tende a ser mais acentuada nesse período. Entretanto, os pacientes operados podem perder até 74% do peso inicial ao longo de 2 anos.^(^^12^^)^Este estudo aportou achados interessantes. Em primeiro lugar, as participantes do grupo IUE eram mais velhas (p=0,024) e tinham circunferência abdominal maior (p=0,048). Para cada ano adicional de idade, o risco de IUE aumentou 11,8% (p=0,009). Cada centímetro de ganho de circunferência abdominal se traduziu em aumento de 5,7% (p=0,05) do risco de IUE antes da cirurgia. A prevalência e a gravidade da incontinência urinária aumentam com a idade. Estudos populacionais revelam uma prevalência de incontinência de 30% a 40% entre mulheres de meia-idade e prevalência mais alta (de até 57%) em mulheres mais velhas.^(^^2^^,^^4^^)^ Entretanto, estudos controlados para outras comorbidades sugeriram que a idade não é um fator de risco independente para incontinência. Níveis elevados de adiposidade central e aumento resultante da pressão intra-abdominal podem ser fatores de risco importantes par a IUE. Por exemplo, a análise combinada do IMC e da circunferência abdominal de 2.702 mulheres com idade entre 42 e 45 anos revelou aumento do risco de prevalência de IUE com cada centímetro de ganho de circunferência abdominal (RC=1,04; IC95%: 1,02-1,06), mas não incrementos unitários do IMC (RC=0,99; IC95%: 0,95-1,04).^(^^5^^)^

Em segundo lugar, a IUE diminuiu de forma significante nos primeiros 6 meses após a cirurgia bariátrica. Nesta coorte, 51,1% das mulheres tinham diagnóstico pré-operatório de IUE. Entretanto, apenas 14% deixaram de atingir a resolução da incontinência 6 meses após a cirurgia (p=0,021), com índice de resolução de 72,7%. Os sintomas urinários também melhoraram, principalmente a IUE (de 51% para 25%; p=0,026) e a noctúria (de 34% para 4%; p=0,001). Os achados deste estudo refletem os relatados por outros autores (índice de resolução da IUE de 80% a 92% após perda significativa de peso).^(^^8^^,^^10^^,^^11^^)^

Em terceiro lugar, a qualidade de vida das pacientes melhorou. Os escores do ICIQ-UI SF diminuíram em 85,3% entre as mulheres que atingiram a resolução (7,68 pontos mais baixos do que entre mulheres que não atingiram a resolução, em média). A perda de peso tem impacto importante sobre a qualidade de vida, e correlações com achados urodinâmicos foram relatadas.^(^^10^^,^^19^^)^ Os escores obtidos no PGI-I e na EVA para satisfação revelaram melhora dos sintomas relatados (p=0,025 e p=0,002), sugerindo melhora do bem-estar geral, não apenas devido à perda de peso, mas também devido a ganhos indiretos, como a resolução da IUE.^(^^14^^,^^15^^)^ O oposto também se verifica: os escores de qualidade de vida/satisfação foram 2,5 pontos mais baixos (p=0,004) entre as mulheres que não atingiram a resolução da IUE.^(^^15^^)^ Portanto, espera-se que a ocorrência da IUE após a perda de peso se traduza em melhora ainda maior da qualidade de vida.

Em quarto lugar, preditores importantes do desfecho da IUE após a perda de peso foram identificados neste estudo. A persistência da IUE mostrou-se significantemente associada à idade, ao parto vaginal e à menopausa (p=0,041, p=0,031 e p=0,029, respectivamente; análise univariada), porém a menopausa foi o principal preditor da persistência da IUE 6 meses após a cirurgia bariátrica (análise multivariada). A persistência da IUE após a cirurgia foi 2,7 vezes mais provável nas mulheres que estavam na menopausa do que nas que não estavam (p=0,046). A menopausa mostrou-se associada a maior risco de IUE. Entretanto, a idade e a menopausa são estreitamente relacionadas, e a idade é um fator bem conhecido para IUE: o risco de IUE é 1,8 vez mais alto em mulheres acima dos 50 anos do que em mulheres de 40 anos.^(^^4^^)^ A persistência da IUE associada à idade e à menopausa após a perda de peso resulta dos baixos níveis séricos de testosterona e da sarcopenia. A perda de peso rápida e excessiva pode causar sarcopenia. A relação entre níveis séricos baixos de testosterona e risco mais elevado de incontinência em mulheres se deve à contribuição da musculatura pélvica para a sustentação da uretra e aos efeitos anabólicos dos androgênios sobre a musculatura esquelética.^(^^20^^,^^21^^)^ A reposição de testosterona pode melhorar o prognóstico das pacientes que sofrem de IUE. Entretanto, a associação entre a IUE e os baixos níveis séricos de testosterona ainda não foi bem estabelecida. A idade também pode afetar a integridade do assoalho pélvico, causando hipermobilidade uretral e deficiência esfincteriana intrínseca, devido à perda de tônus muscular e ao adelgaçamento da mucosa da uretra.

Isso pode levar ao fechamento incompleto da uretra e à perda de urina durante episódios de aumento da pressão abdominal (espirros, tosse etc.).^(^^16^^,^^17^^,^^21^^)^

Finalmente, não foram encontradas correlações entre a magnitude da perda de peso e a IUE pós-operatória neste estudo (p=0,33). A IUE parece ser multifatorial em mulheres obesas. Achados semelhantes foram relatados em estudos prévios com seguimento mais longo.^(^^10^^,^^11^^)^

Este estudo tem algumas limitações. A amostra foi relativamente pequena e o seguimento, curto. Entretanto, os achados encontrados podem ser úteis para dosar as expectativas antes da cirurgia bariátrica e identificar candidatas com maior chance de necessitar de procedimentos adicionais para controlar a IUE.

## CONCLUSÃO

Em conclusão, mulheres mais velhas e com circunferência abdominal maior têm mais risco de desenvolver incontinência urinária de esforço antes da cirurgia bariátrica. Esse tipo de incontinência tende a persistir em mulheres na menopausa, apesar da perda de peso. A perda de peso induzida pela cirurgia bariátrica melhora os sintomas de incontinência urinária de esforço e diminui o impacto sobre a qualidade de vida na maioria das mulheres.
